# Prof. Datuk Dr. Daniel Mahendran Thuraiappah (10 October 1939 to 21 October 2022)

**DOI:** 10.51866/ob.002

**Published:** 2022-11-30

**Authors:** Kaviyarasan Sailin

**Affiliations:** 1 Chairman of Council, Academy of Family Physicians of Malaysia.

On 21 October 2022, Prof. Datuk Dr Daniel Mahendran Thuraiappah, a loving husband, father of two, grandfather and visionary leader passed away at the age of 83 years. He was a great family physician who kept in touch with his patients for five decades and continued to serve the community even after his retirement. It is touching to note that on the eve of his demise, he visited his clinic not knowing that it was going to be his last visit.

He was a doyen of family medicine, keen teacher and pillar with exemplary qualities who rendered endless support to the Academy of Family Physicians of Malaysia (AFPM), the Malaysian Medical Association and general practitioners both locally and internationally.

**Figure:**
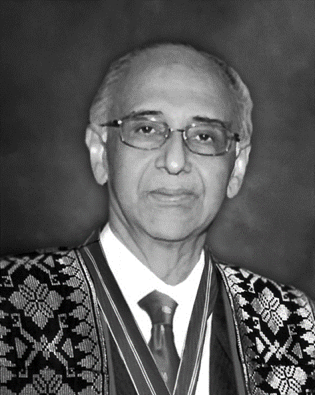


Prof. Thuraiappah, a Malaysian of Ceylonese Tamil origin, studied at Victoria Institution, Kuala Lumpur, and graduated from Queen’s University Belfast with a BSc (Hon) in Anatomy on a bursary award in 1962 and MBBCh BAO in 1965. After his house officer tenure in Belfast, he returned to Malaysia and continued as a medical officer at General Hospital, Kuala Lumpur, until 1969. He also served as a lecturer at the University Malaya Medical Faculty for 2 years.

After serving the government, he opened his clinic, Klinik Thurai, at Sungai Besi, Kuala Lumpur, in 1970 and had been a general practitioner serving the community until his retirement. During his years as a general practitioner, he actively held various posts in the Malaysian Medical Association and later became the chairman of the MMA-PPS. Prof. Thuraiappah also held various posts in Koperasi Doktor Malaysia, where he played a key role in setting up the Info Centre of FOMEMA. He was one of the early general practitioners who embraced digital technology and initiated online continuing medical education.

A passionate teacher, who never stopped learning, enrolled in the Vocational Training Program in Family Medicine. In 1995, he passed the membership examination and was made a member of the AFPM. In 1996, he became a Fellow of the Australian College of General Practitioners. In 1998, he was appointed an adjunct associate professor in family medicine at the University of Putra Malaysia. Prof. Thuraiappah was then awarded Fellowship of the AFPM in 2000 and Honorary Fellowship of the Royal Australian College of General Physicians in 2006. He was also an Honorary Fellow of the College of General Practitioners of Sri Lanka, a Fellow of the Academy of Medicine Malaysia and a Fellow of the Royal College of Physicians, Edinburgh.

Prof. Thuraiappah served the AFPM with great enthusiasm and worked incessantly with the founding fathers to train general practitioners throughout the country. He served as the treasurer, secretary, chairman and finally, president of the academy. He was amongst a few like-minded persons who fearlessly walked the corridors of power at the inception to present the academy’s academic programme to the authorities and was persistent until he gained recognition during his tenure as the chairman of the council. He was at the forefront with a team of committed fellow doctors, working towards recognition of family medicine as a specialty to achieve specialist status, which he eventually succeeded in after many attempts. Prof. Thuraiappah also served as the chairman of the quality improvement committee, promoting quality in general practice/ primary care.

He served as the director of Medibase Sdn Bhd, the e-learning education and training arm of the AFPM. He was instrumental in developing the Diploma in Family Medicine, a 2-year programme aiming to facilitate the qualification of all general practitioners for entry into general practice. He was a long-serving board member of the Faculty of the Diploma in Family Medicine and was instrumental in the development of many short courses of interest for general practice, such as primary care cardiology and ultrasound.

Prof. Thuraiappah enthusiastically promoted the AFPM in various international fora, especially at the World Organization of Family Doctors (WONCA). He served as the vice president of the WONCA Asia Pacific Region, the chairman of the WONCA Working Party on Quality and Safety in Family Medicine, the honorary secretary of the WONCA Asia Pacific Region, a member of the Medical Informatics Group WONCA, a member of the WONCA Bye-Laws Committee and a member of the World Informatics Classification Committee. He was commended for his committed service in the development and promotion of general practice education and quality improvement programmes. Further, he was instrumental in setting up the Asian Regional Primary Care Coalition.

He was the vice chairman of the Joint Land Development Committee of the Academy of Medicine and the AFPM from 2000 to 2010 and a member of the Board of Management of the Building owned by the Academy of Medicine Malaysia and AFPM from 2010 to 2017. After a long development period of 27 years, he, along with the team from the Academy of Medicine of Malaysia and the AFPM, jointly built the Academies Building on the Joint Colleges Land next to the Istana Budaya and the National Library in Jalan Tun Razak, Kuala Lumpur.

Prof. Thuraiappah retired from full-time general practice in 2011 and was appointed an associate professor in family medicine at MAHSA University, Malaysia. In 2013, he was promoted to full professor of family medicine. He had numerous research papers and publications along with many other achievements.

He served as a member of the International Advisory Board of the British Journal of General Practice from 2010 to 2016, a board member of Koperasi Doktor Malaysia and the president and an advisor of the Federation of Malaysian Sri Lankan Organisations for the Malaysian Ceylonese community.

He was a great philanthropist and a community leader who contributed immensely to the redevelopment of the Green Memorial Hospital in Manipay, Sri Lanka.

Prof. Thuraiappah received the following awards for his exemplary public service: Darjah Kebesaran Setia-Sultan Salahuddin Abdul Aziz Shah in 1988, Ahli Mangku Negara in 1989, Kebesaran Dato’ Paduka Setia Mahkota in 2000, Pingkat Jasa Negara-Datuk in 2004, Sri Lankan Excellence Medal in 2007 and Malaysian Medical Association Wilayah Long Service Award in 2022.

He loved music, arts, gardening and his three dogs and one cat.

Prof. Thuraiappah was best known for his kindness, smile, inspiration and generosity. He was a soft-spoken, humble gentleman with an excellent command of the English language, was a person of faith and lived a life serving others. He is survived by his wife Datin Prof. Dr Gnanasothie Duraisamy and their daughter Sothie Naomi and son Daniel Amarasingam.

He will be missed but not forgotten.

